# Clutter elimination for deep clinical optoacoustic imaging using localised vibration tagging (LOVIT)^☆^

**DOI:** 10.1016/j.pacs.2013.07.002

**Published:** 2013-08-02

**Authors:** Michael Jaeger, Jeffrey C. Bamber, Martin Frenz

**Affiliations:** a Institute of Applied Physics, University of Bern, Sidlerstrasse 5, 3012 Bern, Switzerland; b Joint Department of Physics, Institute of Cancer Research and Royal Marsden NHS Foundation Trust, Downs Road, Sutton, Surrey SM2 5PT, UK; c CRUK-EPSRC Cancer Imaging Centre, Institute of Cancer Research and Royal Marsden NHS Foundation Trust, Downs Road, Sutton, Surrey SM2 5PT, UK

**Keywords:** LOVIT, localised vibration tagging, OA, optoacoustic, optoacoustics, epi-OA, epi-optoacoustic, epiphotoacoustic, ARF, acoustic radiation force, PSF, point spread function, FOV, field of view, Photoacoustic, Epiphotoacoustic, Ultrasound, Contrast, Imaging depth, Radiation force, Elastography

## Abstract

This paper investigates a novel method which allows clutter elimination in deep optoacoustic imaging. Clutter significantly limits imaging depth in clinical optoacoustic imaging, when irradiation optics and ultrasound detector are integrated in a handheld probe for flexible imaging of the human body. Strong optoacoustic transients generated at the irradiation site obscure weak signals from deep inside the tissue, either directly by propagating towards the probe, or via acoustic scattering. In this study we demonstrate that signals of interest can be distinguished from clutter by tagging them at the place of origin with localised tissue vibration induced by the acoustic radiation force in a focused ultrasonic beam. We show phantom results where this technique allowed almost full clutter elimination and thus strongly improved contrast for deep imaging. Localised vibration tagging by means of acoustic radiation force is especially promising for integration into ultrasound systems that already have implemented radiation force elastography.

## 1 Introduction

In optoacoustic (OA) imaging, tissue irradiation using pulsed laser light, and subsequent thermo-elastic conversion of absorbed light to ultrasound, allows the detection of optically absorbing structures deep inside biological tissue with high resolution using ultrasound receive beamforming [1], [2], [3]. This technique is especially promising for functional imaging of the vasculature [3], in particular of the oxygenation level based on the different optical absorption spectra of oxy- and deoxyhaemoglobin [4]. OA imaging therefore holds promise for the diagnosis of vascular diseases and cancer [5] and for monitoring response to treatment [6], [7]. In addition, gold nanoparticles, tailored to strongly absorb light in the NIR range, can serve as contrast media [8], [9], and their functionalisation for specific biochemical targets potentially allows early detection of diseases such as cancer [10], [11] and atherosclerosis [12].

The potential of OA imaging as an additional functional imaging modality augmenting classical ultrasound (US) in a real-time, safe, economical, and versatile multimodal device for improved clinical diagnostics has been demonstrated a number of times [13], [14], [15], [16], [17]. For such a combination, an epi-style setup is preferred where the optical components are attached to the acoustic probe for irradiation of the tissue through the same surface where the PA signal is detected. This allows the clinician to guide the combined probe with a single hand while having the other hand free for system operation, and maximises the fluence and thus the SNR in the imaged tissue region. Most importantly, however, the epi-illumination-mode, as opposed to orthogonal or transmission mode, enables imaging of body parts where bones, acoustically attenuating soft tissue (abdomen, limbs, large breast), and gas (abdomen, thorax, neck) would obstruct propagation of acoustic waves from the illuminated tissue region to the acoustic probe.

An important requirement for a clinically successful combination of OA and US imaging is an adequate imaging depth of several centimetres. Such imaging depths are theoretically predicted as feasible, taking into account the optical attenuation and the front-end electronic noise [18]. Such an imaging depth has, however, been difficult to achieve in practise. An important contributing reason is that the epi-OA setup causes severe clutter, which degrades signal-to-background contrast and therefore limits imaging to a depth considerably less than the theoretically possible noise-limited depth, which is dependent on the acoustic centre frequency. As a result, an imaging depth of around one centimetre or even less is typically achieved when using probes that operate in the region of 7.5 MHz [17], [19], [20]. Clutter can emerge from strong OA transients that are generated at the site of tissue irradiation close to the ultrasound probe, where optical absorbers such as melanin and microvasculature are exposed to the highest laser fluence. These transients travel to the acoustic probe on a direct way, generating direct clutter, but also after being scattered by echogenic structures located inside the tissue, causing echo clutter [17], [19]. Either type of clutter can obscure weak signals from deep inside the tissue.

Clinical OA imaging thus requires methods for clutter reduction to achieve the theoretical depth of several centimetres. For this purpose, deformation-compensated averaging (DCA) was previously developed [17], [20], [21]. DCA exploits the clutter decorrelation that results from tissue deformation when slightly palpating the tissue with freehand motion of the imaging probe. Motion compensation of the resulting OA images and subsequent averaging maintains true OA detail but reduces decorrelating clutter similar to stochastic noise. DCA benefits from the combination of OA with US because accurate knowledge of tissue motion can directly be obtained from US speckle tracking.

Evaluation of DCA in combined clinical OA and US imaging of human volunteers has demonstrated that although clutter can significantly be reduced [21], it still has some inherent disadvantages: First, it can only be employed for easily deformable tissue such as the breast and the limb muscles, and it requires considerable practice for controlled probe motion if it is to achieve optimum results. Second, and more importantly, the clutter reduction achievable is limited by the minimum deformation that is required for clutter decorrelation together with the achievable tissue deformation. As a result the number of images with independent clutter typically reduces to around ten, allowing for a maximum contrast gain of about three [20]. A significantly larger contrast gain, however, is necessary to achieve electronic-noise limited imaging depths.

With the aim of overcoming these disadvantages we developed a novel method, localised vibration tagging (LOVIT), which readily overcomes the limitations of DCA and theoretically allows full clutter elimination without the need for tissue palpation. Transient localised tissue vibration, as opposed to the quasistatic deformation in DCA, “tags” the OA signal at the place of origin, allowing the potentially unambiguous identification of this signal and thus full clutter cancellation. Such localised transient vibration can be induced, among other potential methods, by means of the acoustic radiation force (ARF) generated by an ultrasonic focused beam. If technically feasible, using the same transducer for both imaging and transmission of the ARF beam will have the strong advantage that the focus of the ARF beam is inherently aligned with the OA imaging plane. Furthermore, the use of a transducer array will allow steering of the focused beam via the transmit phase of the individual transducer elements for flexible generation of ARF in any location within the imaging plane. ARF generation using transmit beam forming is already implemented in radiation force based ultrasound elastography methods, such as acoustic radiation force impulse (ARFI) imaging and shear wave elastography (SWE), where ARF ultrasound transmission over a fraction of a millisecond generates a localised tissue displacement on the order of a few tens of micrometres [22], [23], [24], [25], [26]

The goal of this paper is to present our first experimental results as a proof-of-principle of ARF-LOVIT. Instead of the imaging probe, a separate transducer, specifically designed for ARF beam transmission, was used. We demonstrate for the first time nearly full clutter elimination and virtually noise-limited epi-OA imaging of tissue-mimicking gelatine phantoms. The ARF-LOVIT results are comprehensively compared to classical epi-illumination OA images that were obtained using the same setup.

## 2 Theory

**Fig. 1:**
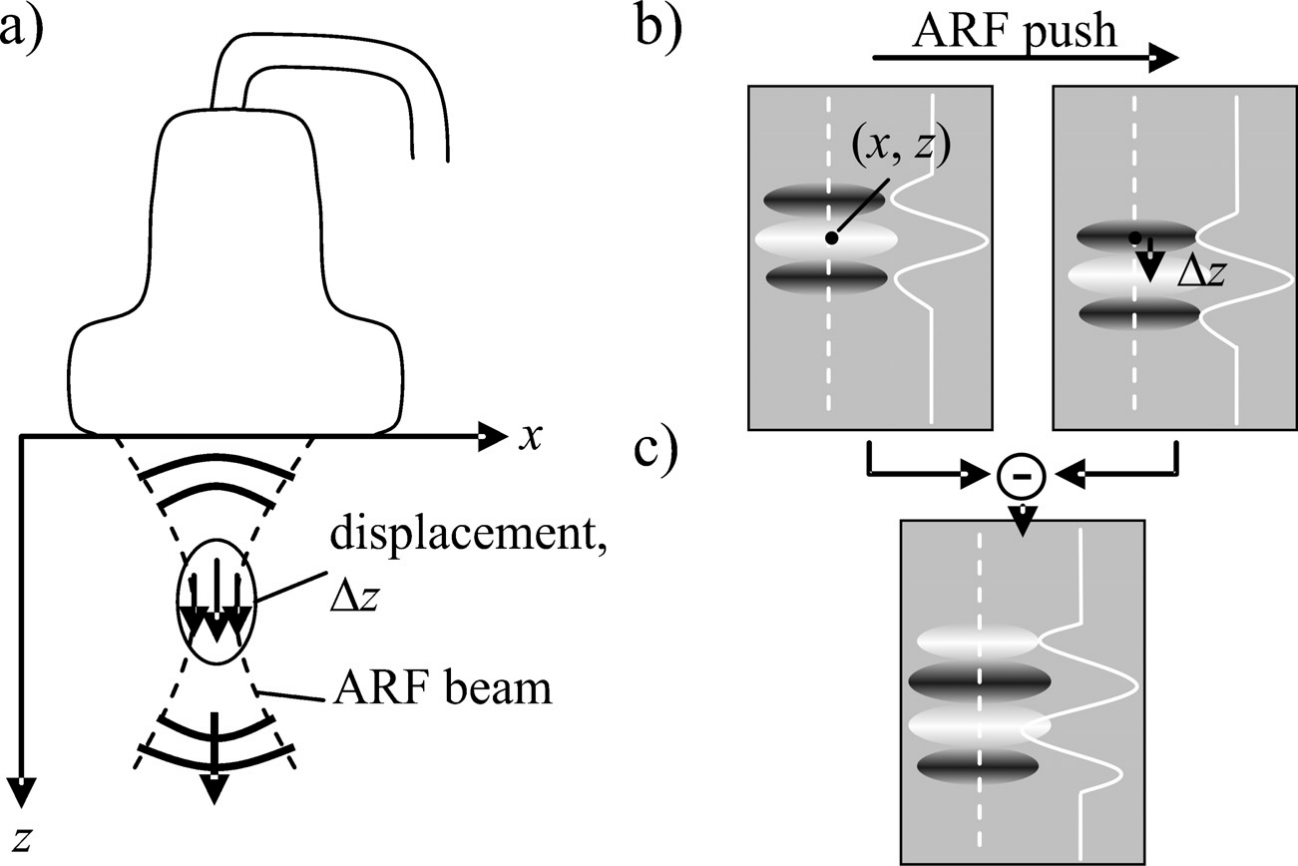
(a) Setup with linear array probe for both imaging and transmission of a focused ultrasonic beam for acoustic radiation force (ARF) generation. (b) The signal from a point absorber is shifted in axial direction when comparing OA images acquired pre and post ARF push. The images show the 2D point-spread function (PSF) originating from the point absorber, as well as the axial profile of the PSF along the dashed lines. (c) In a difference image the point absorber's signal shows up as a slightly changed PSF.

ARF-LOVIT clutter reduction employs localised transient displacement which is remotely induced inside the tissue by transmission of a focused ultrasonic beam (ARF beam) from outside the tissue. In our envisaged mode of clinical implementation of ARF-LOVIT the same linear array transducer will be used both for OA imaging and for ARF beam transmission (Fig. 1a). The high intensity ultrasound in the focus of the ARF beam generates a volumenetric radiation force upon absorption and backscattering. Integration of this force over the duration of the transmission period (few 100 microseconds) results in an impulse transfer to the tissue (ARF push) that initiates a localised tissue displacement of up to several tens of micrometres in magnitude.

A first OA image is acquired prior to the ARF push (i.e. in the absence of displacement at the focus region), and a second one immediately after the push. The spatial extent of the displacement region is determined both by the size of the ARF focus and by the shear wave propagation during the transmission period. Because shear wave speed in tissue is much slower than sound speed, it is possible to make this period short enough so that a narrow and short region of non-zero tissue displacement is still present after ARF beam transmission has already ended. To simplify the theoretical analysis we further assume that: (1) the ARF beam axis runs parallel to the transducers axial direction; (2) the ARF intensity is a function of the coordinates (*x*: lateral direction parallel to the linear array; *z*: axial direction) inside the imaging plane and zero outside; and (3) the local displacement has only an axial component Δ*z*(*x*, *z*) (see Fig. 1).

Firstly we consider a situation without clutter, where a single hypothetical optical point absorber is located at point (*x*, *z*) in the imaging plane and inside the displacement region. The pre-ARF OA image shows the point-spread function (*PSF*), centred at (*x*, *z*) (Fig. 1b, left side). The amplitude *U* is proportional to the local fluence and the absorption cross-section of the point target. The post-ARF OA image shows the same *PSF* but shifted by Δ*z*(*x*, *z*) in axial direction (Fig. 1b, right side). On subtracting the two images a new image is obtained, with a signal occurring at point (*x*, *z*) but with a different point-spread function (*PSF*′) and different amplitude (*U*′) (Fig. 1c). *U*′ and *PSF*′ are determined by Eq. (1):

(1) $$\begin{array}{l} u^{\prime }(x,z)=U\cdot [PSF(x,z-\Delta z)-PSF(x,z)]≅-U\cdot \Delta z\cdot \frac{d}{dz}PSF(x,z)≐U^{\prime }\cdot PSF^{\prime }(x,z)\\ \text{where}\;U^{\prime }≐U\cdot \Delta z\frac{2\pi }{\lambda_{0}}\\ PSF^{\prime }(x,z)≐-\frac{\lambda_{0}}{2\pi }\frac{d}{dz}PSF(x,z) \end{array}$$

To maintain sensible units and magnitudes for both *U*′ and *PSF*′, the acoustic wavelength at the centre frequency of the imaging probe, *λ*_0_, was introduced. In the case of a simple cosine model for the axial profile of the *PSF*, *PSF*′ has equal amplitude as *PSF*. Eq. (1) illustrates that the *PSF*′ of the difference image is in first approximation the axial derivative of the initial *PSF*, and the amplitude *U*′ is proportional to both the initial amplitude *U* and the displacement Δ*z*. The linear approximation to the axial derivative in Eq. (1) holds for Δ*z*/*λ*_0_ < 0⋅5. This assumption is in agreement with in vivo results of radiation force elastography where achievable displacements (on the order of few tens of micrometres, [22], [23], [24], [25]) are far smaller than the wavelength typically used for imaging (e.g. 200 micrometres for a 7.5 MHz centre frequency probe).

**Fig. 2:**
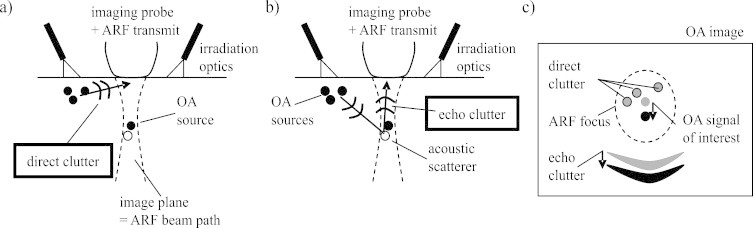
(a) Direct clutter is caused by the strong OA transients that are generated outside the imaging plane and then propagate directly to the imaging probe. (b) Echo clutter is caused by the same OA transients but via acoustic backscattering when they propagate into the tissue. (c) “True” OA signal, direct clutter, and echo clutter are differently influenced by the ARF-induced localised tissue displacement.

Next, clutter is taken into account. Direct clutter emerges from optically absorbing structures that are located outside the imaging plane, but are exposed to the irradiating laser light (Fig. 2a). The resulting strong OA transients, even though detected by the probe at an angle where elevational sensitivity is low, can obscure the weak OA signals from structures deep inside the tissue. Echo clutter (Fig. 2b) may be generated by the same OA transients that cause direct clutter, but via acoustic backscattering at echogenic structures located inside the imaging region. Epi-OA images of the human body usually show both clutter types, and it is impossible to distinguish clutter from “true” OA signal in a conventional image. This often limits the effective imaging depth to less than one centimetre, even if a larger depth would be feasible given the optical penetration depth and the acoustic sensitivity.

ARF-LOVIT allows full elimination of both direct and echo clutter if ideal conditions are met. If the same linear array transducer is used for ARF and imaging, the ARF beam is in elevation confined to the imaging plane. Therefore it does not displace the strong sources of OA transients outside the imaging plane, and direct clutter does not show up in the difference image (see Fig. 2c). Echo clutter on the other hand is caused by acoustic scattering from within the imaging plane. Echoes generated inside the displacement region are shifted in agreement with the underlying echogenic tissue structures, leading to residual echo clutter in the difference image (see Fig. 2c). However, the ultrasound that ultimately leads to echo clutter propagates through the tissue twice, from the region where a strong OA transient is generated to the echogenic structure and back to the acoustic receiver. Therefore residual echo clutter shows up at a different depth in the difference image than the OA signals coming from the displacement region. Provided the axial extension of the displacement region is small enough, direct OA signals and echo clutter are spatially separated, and the difference image shows OA signals from inside the displacement region free of both direct and echo clutter.

This section illustrates that the difference image, henceforth called “LOVIT image”, shows an OA image of the inside of the displacement region without clutter. The original amplitude is compounded with the spatial distribution of the displacement Δ*z*(*x*, *z*), and the PSF is slightly changed. The original amplitude can in principle again be obtained by dividing by Δ*z* (and, in theory, the original PSF by spatial integration). By scanning the imaging plane with the ARF focus, a clutter-free composite LOVIT image can thus be generated of the full imaging plane which conserves the true OA signal amplitude, but eliminates clutter. The conservation of absolute signal amplitude by LOVIT is important in view of data analysis that relays on accurate amplitude, such as blood oxygen saturation imaging.

## 3 Materials and methods

The goal of the experimental study was the proof of principle of clutter elimination using ARF-LOVIT. In a preferred mode of implementation of ARF-LOVIT the same transducer is used both for ARF beam transmission and for imaging. This is technically feasible, and already commercially implemented for radiation force elastography [23], [27]. However, this study used a separate transducer for ARF beam transmission, in conjunction with a commercial ultrasound scanner for imaging.

### 3.1 Equipment and setup

The commercial ultrasound scanner (z.one™ from Zonare Medical Systems Inc. USA), in conjunction with a linear array probe (L10-5, Zonare), was operated in a dedicated research mode which facilitated parallel readout and storage of channel data from a subaperture of 64 elements out of the 128 element array. This allowed the acquisition of an OA frame with 19 mm aperture and several centimetres depth after each laser pulse, and storage of long frame sequences (up to minutes at the laser pulse repetition rate of 10 Hz) on internal memory for subsequent read-out. The L10-5 featured a bandwidth (−3 dB) of 5–10 MHz and 7.5 MHz centre frequency corresponding to an acoustic wavelength of 200 μm. For OA signal generation, a Q-switched Nd:YAG laser (ELEN, Italy) was used delivering 70 mJ per pulse at 1064 nm wavelength with a 7 ns pulse duration and 10 Hz repetition rate. The laser light was guided via a bifurcated fibre-optic bundle (Fibreoptic, Switzerland) through two profile converters which were attached together and generated a single line of irradiation of 20 mm length and about 5 mm width parallel to one long side of the aperture of the linear probe.

Besides OA imaging, the z.one was used for additional purposes: First, it allowed the acquisition of conventional pulse-echo US images of the investigated phantoms for comparison of the OA images with “anatomical” features seen in US. Second, pulse-echo channel data could be acquired at a high framerate of 2 kHz for characterising the displacement magnitude in the ARF focus and of the subsequent shear-wave propagation using correlation-based tracking of the axial phase [28]. This was important in view of interpretation of the LOVIT results.

**Fig. 3:**
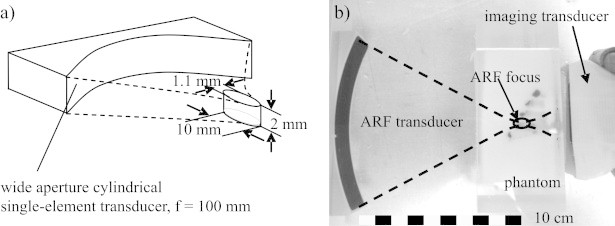
(a) Sketch of the ARF transducer and dimensions of its focal region. (b) Detail view of the experimental setup showing the ARF transducer and the imaging probe, together with a phantom. The focused ARF beam is indicated by dashed lines.

For ARF beam transmission a separate, custom-made, cylindrically shaped single-element transducer was used. The size of the aperture was designed to be large in the direction of the curvature (100 mm), but narrow in direction parallel to the cylinder axis (Fig. 3a). This provided a flat focused beam with a focus that was narrow in the lateral dimension of the beam plane, but comparably wide in elevation. This ARF transducer could be scanned in a plane using two motorised linear stages (T-LLS105, Zaber), and the imaging array was aligned opposite to the ARF transducer, such that the imaging plane matched the ARF-beam scanning plane. Fig. 3b shows a detail view of the setup with the ARF transducer and the imaging transducer inside a water tank. The size and location of the phantom is also seen and the ARF focal position inside the phantom is indicated. The ARF transducer was driven at its centre frequency (2 MHz) using a waveform generator (33220A, Agilent) via an RF-amplifier (Tomco).

### 3.2 Tissue phantom

**Fig. 4:**
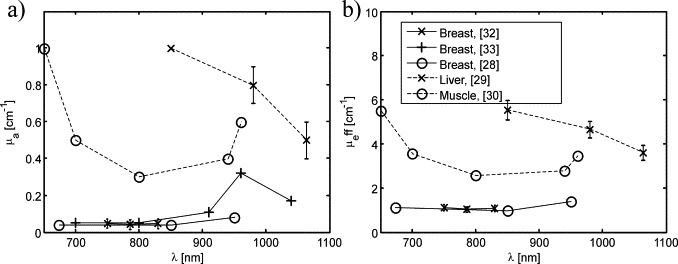
Tissue optical properties for human breast, liver, and skeletal muscle. (a) Absorption coefficient. (b) Effective attenuation coefficient.

The tissue phantoms were intended to mimic optical properties of human tissue in the NIR range which is preferred for deep OA imaging (diagnostic window). Fig. 4 summarises optical properties of various human tissue types in the range from 650 nm to 1100 nm, quoted from [29], [30], [31], [32], [33], [34]. The effective optical attenuation coefficient of the bulk tissue determines the depth-dependent OA signal level whereas the absorption coefficient at the tissue surface determines the level of direct clutter, and, together with the tissue echogenicity, the echo clutter level. Therefore optical attenuation, optical absorption and acoustic echogenicity together determine the clutter-limited imaging depth. In addition to the optical properties the phantom's elasticity had to be similar to that of tissue.

In order to meet all these criteria the phantoms were built from gelatine for elasticity (Fluka analytical, from porcine skin), TiO_2_ for optical scattering (Sigma Aldrich), India ink for optical absorption (951 black Winsor & Newton), and cellulose for echogenicity (Sigmacell Type 20). Cellulose is a popular agent for acoustic scattering in ultrasound phantoms, with concentrations (weight) ranging from 0.25% [35] to several percents [36]. To assess the effect of LOVIT on contrast and imaging depth the phantoms contained 2-mm-diameter gelatine cylinders with a larger optical absorption coefficient than the background, mimicking blood at around 800 nm. These artificial blood vessels were on purpose made hypoechoic (no cellulose) so their true position could be identified on B-mode US.

Three different phantoms were built with slightly differing optical properties of the background. The compositions of the phantoms, as well as key optical properties, are listed in Table 1. The absorption coefficient of the inclusions was estimated based on a priori photometric measurement of the India ink. The bulk effective optical attenuation coefficient *μ*_eff_ at the wavelength used for the experiments (1064 nm) was determined a posteriori based on the depth dependent amplitude of the OA signal of the absorbing inclusions. The resulting *μ*_eff_ of the different phantoms was located between the *μ*_eff_ of breast and muscle tissue in the wavelength range between 800 and 950 nm. Therefore our LOVIT results are also representative for such tissues in the same wavelength range although the wavelength used in the experiment was 1064 nm.

**Table 1:** Composition and key optical properties of the phantoms.

	Gelatine	TiO_2_	India ink	Cellulose	*μ*_eff_	*μ*_abs_
Inclusions	5%	2‰	0.84‰	–		5 cm^−1^ ± 0.1 cm^−1^ (ink)
Phantom I	5%	2‰	–	2%	>1.7 cm^−1^ ± 0.2 cm^−1^	0.17 cm^−1^ (H_2_O)
Phantom II	5%	1‰	–	2%	1.25 cm^−1^ ± 0.2 cm^−1^	0.17 cm^−1^ (H_2_O)
Phantom III	= Phantom II, but with a thin layer of inclusion mixture added on top to mimic optical absorption in the skin				The absorption in the thin layer was smaller than 30%, based on the amplitude ratio of the inclusion signal with and without layer	

Optical absorption by the melanin in the skin layer has been shown to cause prominent echo clutter in clinical epi-OA imaging of the breast of dark-skinned human volunteers (data not yet published). To investigate this effect in our LOVIT phantom experiments, an absorbing surface gelatine layer was added to phantom II, resulting in phantom III (see Table 1). The absorption of this surface layer was estimated by comparing the OA signals from the inclusions obtained with and without this layer.

### 3.3 ARF-LOVIT acquisition procedure

For characterisation of the magnitude and spatial extent of the ARF-induced localised displacement and of the subsequent shear wave propagation, the z.one was operated in the research mode for acquisition of pulse-echo RF channel data. An internal trigger of the z.one triggered the acquisition of a sequence of 30 pulse-echo frames at 2000 fps frame rate (total 15 ms duration). One ms after the first frame, an ARF beam was transmitted for 0.5 ms duration. After ARF beam transmission, the time-dependent local displacement inside the phantom could be observed in the pulse-echo RF frames. For this purpose pulse-echo frames were reconstructed offline using a frequency-domain synthetic aperture algorithm [37]. The local displacement was determined using correlation based axial tracking of the RF echo phase [28] resulting in a movie of the local displacement after the ARF push and of the subsequent shear wave propagation. From this movie, both the magnitude of the displacement and the shear wave were determined.

For the ARF-LOVIT experiment, the z.one was operated in the research mode for RF channel data acquisition, with ultrasound transmission inactive but with the laser active. OA frames were reconstructed using a frequency domain algorithm [38]. A collection of 20 OA frames was acquired without preceding ARF push (reference frames), and a collection of 10 OA frames with preceding ARF pushes (post-ARF frames) where the delay between a push and a frame was one millisecond. In addition to that, 20 frames without laser irradiation were acquired to characterise the stochastic noise level. A single conventional OA image was then obtained from averaging the 20 reference frames. A single pre-ARF frame and a single post-ARF frame were obtained averaging 10 out of the reference frames and the 10 post-ARF frames, respectively. A single LOVIT image was then calculated taking the difference of the two images, multiplied by a factor of 0.5. In that way both the conventional OA image and the LOVIT image were obtained from the same number of acquisitions and exhibited the same stochastic noise level.

This procedure provided a LOVIT image from inside the spatially confined displacement region around a single ARF focus position. In order to obtain a large field of view, the imaging plane was scanned with the ARF focus in 2 mm lateral and 5 mm axial steps. For each focal position a separate LOVIT image was recorded following the image recording procedure described above. Then a composite LOVIT image was generated by mosaicking from all focus positions. For this purpose, all the LOVIT images were compounded with a Gauss profile (with FWHM 2 mm in lateral and 5 mm in axial direction) centred at the respective focus positions, and then summed. This procedure has a slight influence on the local signal-to-noise ratio, and was therefore applied both to generate a composite LOVIT image *and* to generate a compound conventional OA image for fair comparison of the two methods (see Section 4).

### 3.4 Data display

The field of view (FOV) of the reconstructed frames was 19 mm (lateral) by 50 mm (axial) for the fast pulse-echo mode frames and thus for the displacement snapshots, and 38 mm (lateral) by 50 mm (axial) for the OA images. The lateral extent of the FOV for the OA images was chosen deliberately larger than the extent of the receiving aperture (19 mm) for two reasons: First, this allowed simpler comparison of the OA images with the z.one's conventional pulse-echo images which covered the same size. Second, this accounted for the possibility of receive angles pointing outside the axial projection of the active aperture. For display, B-mode OA images were obtained using envelope detection and logarithmic compression. All displayed B-mode OA images cover the same amplitude range of 40 dB, starting at an identical level.

## 4 Results

### 4.1 Combined pulse-echo and conventional OA imaging

**Fig. 5:**
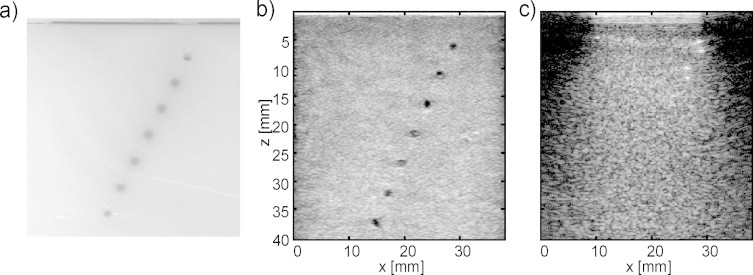
(a) Photograph of a section through phantom III at the location of the imaging plane, taken after the experiment. The intersection areas with the cylindrical inclusions are visible as well as the absorbing top layer. (b) B-mode ultrasound of the phantom, showing the hypoechoic inclusions. (c) Conventional OA image, showing only the most superficial two inclusions whereas deeper inclusions are obscured by clutter.

Fig. 5a shows a photograph of a section through phantom III showing the plane that was imaged during the experiments. The imaging plane was aligned perpendicular to the cylindrical inclusions, and the cross-sections of the inclusions can be nicely seen as circular grey areas arranged along an oblique line. Fig. 5b is the z.one B-mode ultrasound image obtained at the same position, which allows the identification of the optically absorbing inclusions as hypoechoic regions inside the echogenic background. Fig. 5c is the conventional OA B-mode image. This image represents the state-of-the-art of epi-OA imaging without clutter reduction. In Fig. 5c, only the most superficial two inclusions can clearly be identified. The reason for this is clutter, which obscures the deeper inclusions and limits imaging depth. The cross-section of each vessel appears as a double-dot structure on the OA B-mode image. This appearance is the combined effect of the limited bandwidth and of the limited aperture of the ultrasound probe. The bandwidth acts like a spatial band-pass filter highlighting only the vessel boundary, and the limited view of the probe aperture allows only the upper and the lower boundary of the vessel to be detected.

### 4.2 Shear wave and displacement characterisation

**Fig. 6:**
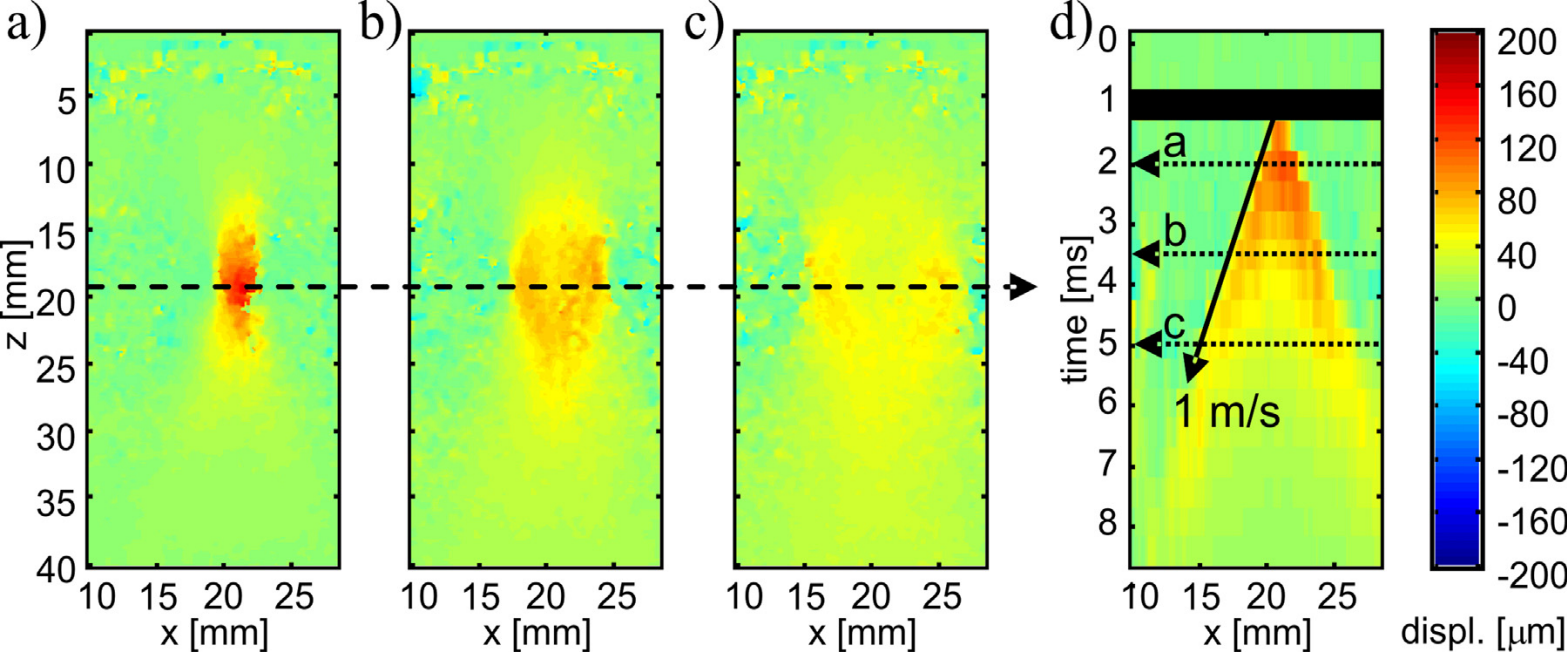
(a) to (c) Snapshots of shear wave propagation after the ARF push at different points of time. (d) Local displacement as function of time at constant depth indicated by the dashed horizontal arrow through (a) to (c). The times at which the snapshots were taken are indicated in (d) by dotted horizontal arrows. The ARF beam transmission period is indicated with a horizontal black bar. The slope of the shear wave propagation, indicated with a solid arrow, allowed the determination of the shear wave speed.

The magnitude of the ARF-induced localised displacement as well as the spatial extension of the displacement region are important parameters for the performance of ARF-LOVIT and were assessed in a first experimental stage. For this purpose the ARF focus was centred in the FOV, and a fast pulse-echo sequence was acquired and analysed. Fig. 6 displays a sequence of displacement snapshots taken at different times after the ARF push, and the displacement along a line of constant depth as function of time. The time evolution shows the localised displacement immediately after the end of the ARF push and the subsequent generation and propagation of transient shear waves. Note that all displacements are positive, although the ARF push acted in negative axial direction towards the imaging probe. This is a mere convention. The temporal slope of the shear wave propagation allowed the determination of the shear wave speed to 1 m/s ± 0.1 m/s. Based on the time evolution of the displacement, the post-ARF acquisition delay was chosen to be 1 ms for the subsequent LOVIT experiment. The achieved displacement magnitude was 130 μm, and the size of the displacement region was roughly 2 mm (laterally) by 5 mm (axially). These dimensions also determined the scanning stepsize for the generation of the large FOV composite LOVIT image.

### 4.3 ARF-LOVIT, single push location

**Fig. 7:**
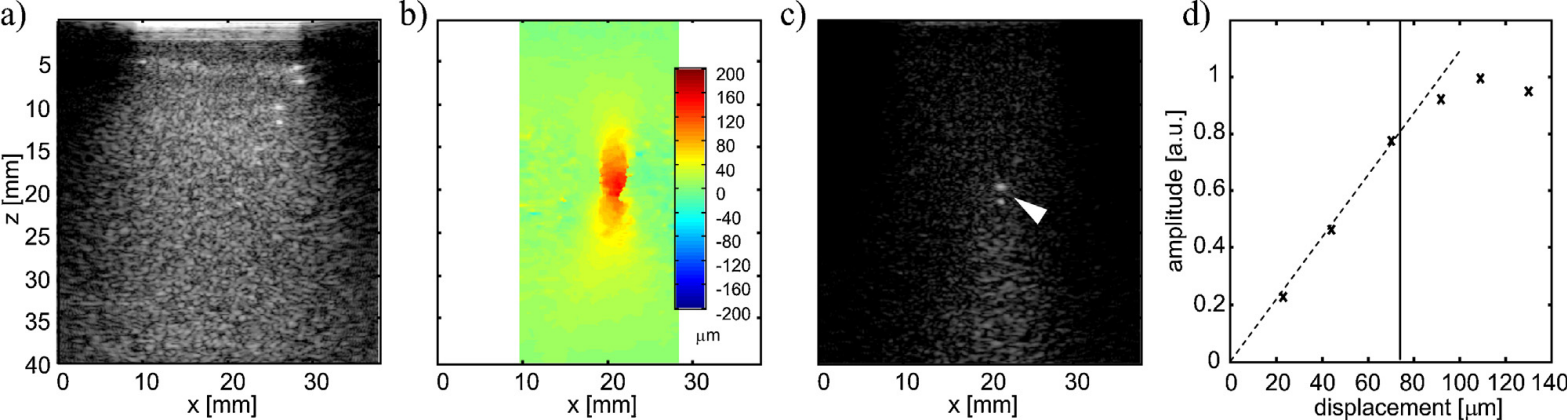
(a) Conventional OA image. (b) Localised displacement after ARF push. (c) Resulting LOVIT image, with fourth inclusion indicated by white arrowhead. (d) Dependence of LOVIT amplitude on displacement magnitude. The dashed line indicates the linear relation between amplitude and displacement for a displacement up to around 75 μm denoted by the solid vertical line.

In a second experimental stage, LOVIT clutter reduction was demonstrated with a single ARF focus position. Fig. 7a is the conventional OA image of phantom III. As previously mentioned, only the most superficial two inclusions could clearly be identified. The ARF focus was then positioned in the centre of the imaging plane where the fourth inclusion was known to be located based on the B-mode US (Fig. 5b). Fig. 7b shows the resulting localised displacement 1 ms after the ARF push, the time of post-ARF acquisition in this experiment.

The resulting LOVIT image, Fig. 7c, shows strongly improved contrast compared to the conventional image, leading to the visibility of the fourth inclusion which was located inside the displacement region. Posterior to the ARF focus position, the difference image shows a region of diffuse signal which extends down to the bottom of the image. This is presumably echo clutter, which emerges from acoustic backscattering inside the displacement region but turns up in the difference image at a larger depth owing to the longer acoustic round-trip time. The OA transients that result in echo clutter originate from distributed sources and thus give the echo clutter region a large spatial extension.

Fig. 7d shows the amplitude of the OA signal in the difference image (LOVIT amplitude) as a function of the localised displacement magnitude. For this measurement the ARF beam transmission period was changed from short to long to generate different displacement magnitudes. The result confirms the linear relation between displacement and LOVIT amplitude for displacements up to 75 μm, as shown in Eq. (1). The LOVIT amplitude achieves a maximum at around 100 μm displacement corresponding to half the acoustic wavelength at the imaging centre frequency of 7.5 MHz, and then decreases again as destructive superposition starts to occur. At the maximum LOVIT amplitude, the same SNR is obtained as would be achieved with simple averaging over the identical number of frames, but with clutter virtually eliminated around the absorbing inclusion.

### 4.4 ARF-LOVIT, 2D scan

**Fig. 8:**
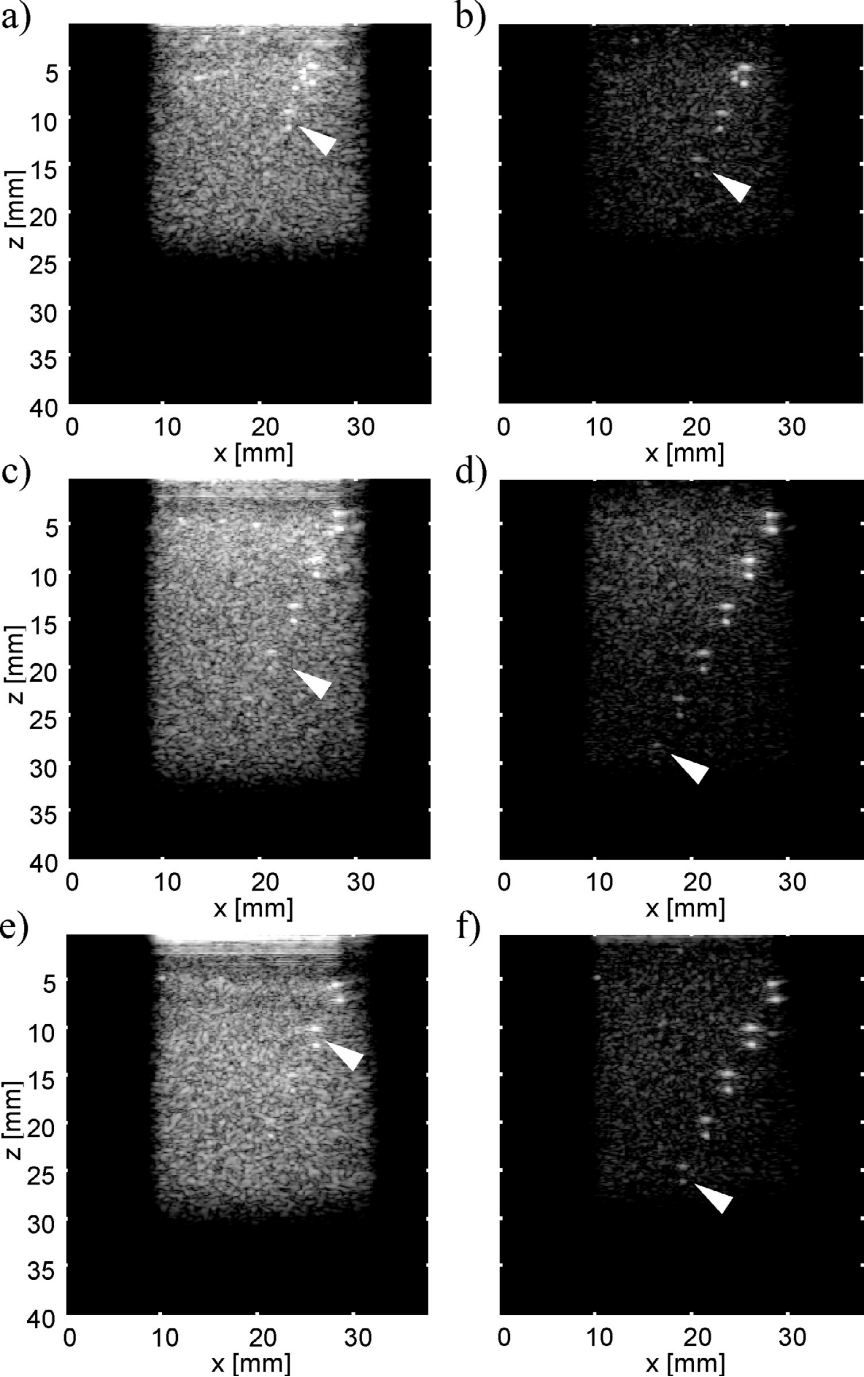
Composite LOVIT results (b, d, f) of the three phantoms, compared to the conventional OA images (a, c, e). (a) and (b) Phantom I. (c) and (d) Phantom II. (e) and (f) Phantom III. The conventional images were generated using the same mosaicking method as used to produce the composite LOVIT images. Since the ARF focus scanning range was different for the three experiments, a different image depth was obtained.

The LOVIT result from a single ARF focus position demonstrated that, within the localised displacement region, clutter can be largely eliminated and thus contrast of true OA signals strongly improved. In a third experimental stage, to demonstrate a process for achieving a large-FOV clutter free image, the ARF focus was 2-dimensionally scanned over the phantom, in steps of 2 mm laterally and 5 mm axially. A composite LOVIT image was then generated by mosaicking as explained in Section 3. The results for the different phantoms are shown in the second column of Fig. 8. For fair comparison, the same mosaicking procedure as for the LOVIT composite image was employed to generate the conventional OA images. These results are shown in the first column of Fig. 8.

All three examples demonstrate both improved contrast *and* increased imaging depth when using LOVIT compared to conventional OA imaging. The residual background signal of the LOVIT image is a combination of system noise, residual echo clutter (see Section 5), and true inhomogeneous optical absorption in the imaging plane. The difference between noise (determined from the noise frames, see ARF LOVIT acquisition sequence) and residual background was around 1–2 dB at large depths where true inhomogeneous absorption was negligible. This indicates that residual echo clutter made up for only 1–2 dB, or 10–25%, of the residual background level and thus virtually noise-limited imaging depth was achieved. Limited by the residual background, the deepest visible inclusion that can unambiguously be identified is indicated for each image in Fig. 8 (white arrowhead). The imaging depth obtained with LOVIT, as well as the clutter-limited conventional imaging depth, is listed in Table 2 for the different phantoms. The theoretical imaging depth limited by only system noise is also listed, extrapolated based on the slope of the depth-dependent LOVIT amplitude of the inclusions, and on the noise level. An SNR of 10 dB (3*σ* significance level) was assumed as threshold for inclusion detection, in agreement with the detection threshold we observed in visual inspection of the conventional OA images.

**Table 2:** Imaging depth obtained with the different phantoms and the different methods.

	Conventional OA imaging depth (mm)	LOVIT (mm)	Noise-limited imaging depth (mm)	Increase in imaging depth (mm)
Phantom I	10–15	15–20	22	5
Phantom II	15–20	>30	30	10–15
Phantom III	10–15	>25	30	15–20

### 4.5 Characterisation of effective attenuation coefficient

Independent experiments have shown that the reconstructed signal amplitude of cylindrical OA sources depends only minimally on the source-transducer distance. With the cylindrical sources, no acoustic diffraction occurred in elevation, and a constant receive angular aperture made sure that acoustic diffraction in lateral direction was compensated for by reconstruction beamforming. Therefore the effective optical attenuation coefficient of the different phantoms could be derived a posteriori from the slope of the depth-dependent OA amplitude of the inclusions. For this purpose we assumed that the local fluence and thus the OA amplitude of the inclusion could be modelled by the diffusion approximation solution for a line source [39], taking into account that irradiation was not homogeneous but occurred on a single line adjacent to the linear probe. According to this model and the data, the effective attenuation coefficient *μ*_eff_ was 1.7 cm^−1^ for phantom I, 1.4 cm^−1^ for phantom II, and 1.1 cm^−1^ for phantom III. For phantom I, only amplitude measurements close to the surface where available, which might have lead to an underestimation of *μ*_eff_ owing to the boundary conditions. Because the bulk material of phantom II and III was identical, we assume an average value of 1.25 cm^−1^ for both. The standard error was around 0.2 cm^−1^.

## 5 Discussion

The results demonstrate that ARF-LOVIT facilitates strongly improved contrast and imaging depth in a situation where OA contrast is limited by clutter rather than by system noise. With various phantoms mimicking optical properties in the range between human breast and muscle tissue, imaging depth could be increased close to the noise limit, suggesting that almost full clutter elimination was obtained with ARF-LOVIT. The largest increase in imaging depth was obtained with phantom III, from 10 mm to at least 25 mm, more than doubling the conventional imaging depth. The smaller conventional imaging depth in phantom III as compared to phantom II is explained with the higher echo clutter level owing to the absorbing “skin” surface layer. Our results indicate that LOVIT allows full clutter elimination and thus noise-limited imaging independent of melanin content. This will especially be important for patients with high melanin content, who might else be excluded from deep OA imaging.

In addition to epi-OA imaging, LOVIT has potential for clutter reduction in echo ultrasound and in optical coherence tomography. Similar problems of clutter-limited image contrast exist in conventional US echography. Acoustic clutter may, for example, arise from acoustic scatterers interacting with side lobes or grating lobes, which may generate clutter echoes that return to the acoustic receiver either directly or after being scattered by other echogenic structures, or reverberation of ultrasound between acoustic scatterers that are proximal to the depth of interest [40]. Approaches that are similar to DCA have been developed for US pulse-echo imaging independently and apparently without awareness of those developed for OA imaging, and perform with similar limitations to DCA in OA imaging [41]. LOVIT can facilitate clutter elimination in US pulse-echo imaging without those limitations. In OCT, loss of contrast with growing depth due to the strong and multiple optical scattering by tissue may be regarded as optical clutter. In the most common form of OCT this is substantially reduced by the use of a highly collimated beam of light [42], however, this does not fully remove the possibility of optical clutter generation at depths where the beam has been diffused by scattering. Scanning an ultrasound beam confocally with the laser beam, LOVIT might further increase OCT imaging depth.

In a clinical application of LOVIT, tissue motion can potentially influence the outcome of clutter reduction. Especially the pulsating arteries, which are one of the main targets in OA imaging, lead to quick motion of the vessel wall and of the surrounding tissue. As long as the tissue motion is fairly localised to the artery, this could even be an advantage, by enabling LOVIT without requiring ARF. In any other cases where tissue motion is not localised, ARF-LOVIT will still be possible provided that the time delay between pre- and post-ARF OA frames is short enough, such that tissue motion in-between the two frames is minimal. LOVIT performance may even be optimised by motion compensation prior to subtracting the tagged and the reference OA images. For this purpose, pulse-echo acquisitions that occur shortly before each pre-ARF frame can be used for motion tracking, and tissue motion can be extrapolated from a multiple of such pulse-echo frames to the time of the post-ARF OA acquisition. Such strategies for avoiding motion artefacts will be investigated in a future study.

**Fig. 9:**
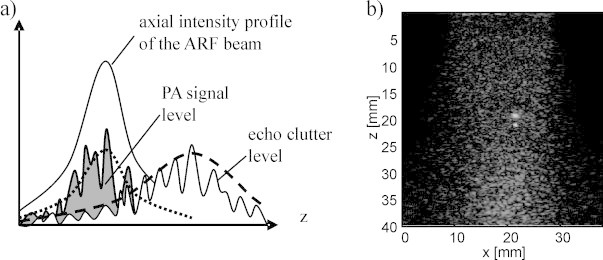
(a) Sketch for the overlap of OA signal and residual echo clutter in the LOVIT image. The depth-dependent envelope of the signal level of the LOVIT OA signal is indicated by a dotted line, and is proportional to the displacement magnitude and thus to the depth profile of the intensity of the ARF beam. The envelope of residual echo clutter is indicated by a dashed line, and is proportional to a stretched version (by a factor of two) of the depth profile of the displacement magnitude. Because the intensity of the ARF beam is non-zero along the whole depth (*z*) axis, the two envelopes intersect, and some residual echo clutter is present at the location of the ARF focus. (b) LOVIT image obtained by subtracting two post-ARF OA frames with adjacent ARF focus positions. Clutter reduction performance is equivalent to the case where a pre-ARF reference frame was used.

Two factors contributed to the extraordinary performance of LOVIT in the presented proof-of-principle study: First, a fairly large localised displacement magnitude, in the range of half the acoustic imaging wavelength, allowed maximum LOVIT amplitude. Second, and more important, a small displacement region allowed the substantial elimination of echo clutter. For full elimination of direct clutter, tissue displacement should be confined to an elevationally bounded region. This condition was met because the transmitted ARF beam was confined to the imaging plane, and the post-ARF image was acquired before the shear wave had spread too far. Echo clutter elimination on the other hand typically requires a displacement region that is particularly well-confined in the axial direction, to allow spatial separation of true OA signal and echo clutter that both originate from the same region. Generally, this requirement cannot be fully satisfied because significant displacement is generated all along the ARF beam path even with a focused beam, leading to an overlap of OA signals and echo clutter in the difference image. This is illustrated in Fig. 9. LOVIT amplifies OA signals proportional to the displacement Δ*z*(*x*, *z*) determined by the ARF beam profile. At the same time, it amplifies echo clutter at larger depth, approximately proportional to a stretched profile, related to but not necessarily equal to Δ*z*(*x*, *z*/2), due to the extended round-trip time of the echoes relative to direct OA signal. Because Δ*z*(*x*, *z*) is not perfectly confined in axial direction, the two profiles overlap resulting in residual echo clutter in the LOVIT image at point (*x*, *z*). For this reason the axial length of the displacement region should generally be kept as short as possible. This was achieved in the present study by a small axial extension of the ARF focus on one hand and, on the other, by taking advantage of the slow shear wave propagation during ARF beam transmission and during the post-ARF acquisition delay.

In clinical practice, both a large displacement and a tightly confined displacement region can potentially be problematic and partially exclusive. Displacement magnitudes that are typically obtained in human tissue with radiation force elastography are situated in the range of 5–40 μm, obtained with ARF beam transmission periods between 100 and 500 μs [22], [23], [24], [25]. The maximum permissible mechanical index (MI) limits the acoustic peak intensity of the ARF beam, thus larger displacements can only be achieved with a longer ARF beam transmission period. Because shear wave propagation already occurs during ARF beam transmission, a longer period above a certain threshold comes with a larger displacement region which in turn reduces the ability of echo clutter cancellation.

For the above reasons, in the case where echo clutter prevails over direct clutter, a tightly confined displacement region with moderate displacement magnitude (e.g. around 20 μm) might be preferable compared to a larger but less localised displacement.

If a moderate displacement magnitude is preferred in view of efficient echo clutter reduction, the achievable SNR of ARF-LOVIT (SNR_lovit_) compared to conventional OA imaging assuming no clutter and averaged over the same number of acquisitions (SNR_conv_) is of interest (SNR gain G). Eq. (2) yields a useful relation between the displacement and *G*:

(2) $$\Rightarrow G≐\frac{{\text{SNR}}_{\text{lovit}}}{{\text{SNR}}_{\text{conv}}}=\frac{U'/\sqrt{2}}{2U/\sqrt{2}}=\frac{U\cdot \Delta z\cdot 2\pi /\lambda_{0}}{2U}=\frac{\Delta z\cdot \pi }{\lambda_{0}}$$

For Δ*z* = 20 μm and *λ*_0_ = 200 μm, the SNR gain becomes *G* = *π*/10. This means that with displacements typical for radiation force elastography, the SNR of the OA image is reduced by a factor of three by ARF-LOVIT compared to simply averaging. If the clutter level is the main factor limiting deep imaging, then such a reduction in SNR is acceptable given the benefit of clutter elimination.

A preference for a short displacement region, for successful echo clutter reduction, may set a limitation to the real-time capability of LOVIT: A short displacement region goes hand in hand with a large number of ARF focus positions for a full FOV composite LOVIT image. With a focal size of the displacement region of 2 mm laterally and 5 mm axially, the minimum amount of acquisitions required to cover a FOV of 20 mm by 40 mm is 80 post-ARF frames plus one reference frame. If motion artefacts are to be avoided, 80 reference frames, acquired directly before the ARF push, are preferable. This would result in 160 acquisitions, or 16 seconds acquisition time, at 10 Hz laser pulse repetition rate, for a single composite image. If this is a problem, lasers with a higher pulse repetition rate can be used to increase acquisition speed.

On the other hand, the total number of acquisitions for a single composite image, and thus acquisition time, can be reduced by employing more sophisticated acquisition and data processing schemes than the one used for this proof-of-principle study. In a first step the number of acquisitions can be reduced by a factor of two because the pre-ARF reference frames are obsolete. Post-ARF frames obtained with spatially separate focal zones can serve as respective reference frames. Fig. 9b shows the result of this approach for phantom III, for two focal zones adjacent to each other, around the position of the absorbing inclusion already shown in Fig. 7c. The same contrast improvement as in Fig. 7c is observed.

A high acquisition rate might ultimately conflict with ultrasound safety because the rate of ARF beam transmissions is limited by the maximum permissible average ultrasound intensity. If this is the case, the total number of ARF beam transmissions can potentially be reduced by taking advantage of shear wave propagation, and acquiring multiple OA frames after a single ARF push.

Note that the above potential limitations to real-time capability count only for echo-clutter. Direct clutter can always be eliminated owing to the narrow localisation of the ARF beam to the imaging plane, even with an elongated ARF beam focus. In summary, real-time feasibility and achievable SNR are a challenge for clinical application of LOVIT which requires further investigation. In comparison to clutter reduction using DCA, LOVIT shows several advantages: The transient localised tissue displacement as compared to static deformation allows elimination of direct clutter as well as echo clutter and thus improved imaging depth; the remote generation of tissue displacement makes the method applicable with non-palpable tissue; the automated generation of tissue displacement using radiation force requires no special skills of the medical practitioner. Future research has to focus on finding the optimum set of acquisition schemes, acquisition parameters, and data processing, for clinically successful LOVIT.

## 6 Conclusion

Localised vibration tagging allows clutter elimination in epi-optoacoustic imaging of phantoms that mimic tissue optical and acoustic properties. In this proof-of-principle study, almost full clutter elimination was demonstrated to be feasible when using acoustic radiation force in the focus of an ultrasonic beam.

## Conflict of interest statement

The authors declare that there are no conflicts of interest.

## Funding sources

This research was supported in parts by the Swiss National Science Foundation (No. 205320-103872), by the Cancer Research UK, and by the Engineering & Physical Sciences Research Council Cancer Imaging Centre grant. M. Jaeger is funded by the SNSF Ambizione grant PZ00P3_142585. We specially thank Zonare Medical System Inc., USA, for providing equipment and technical support.
